# Subgingival bacterial colonization profiles correlate with gingival tissue gene expression

**DOI:** 10.1186/1471-2180-9-221

**Published:** 2009-10-18

**Authors:** Panos N Papapanou, Jan H Behle, Moritz Kebschull, Romanita Celenti, Dana L Wolf, Martin Handfield, Paul Pavlidis, Ryan T Demmer

**Affiliations:** 1 Division of Periodontics, Section of Oral and Diagnostic Sciences, College of Dental Medicine, Columbia University, New York, NY, USA; 2 Department of Epidemiology, Mailman School of Public Health, Columbia University, New York, NY, USA; 3 Center for Molecular Microbiology and Department of Oral Biology, University of Florida, College of Dentistry, Gainsville, FL, USA; 4 Department of Psychiatry and Center of High-Throughput Biology, Michael Smith Laboratories, University of British Columbia, Vancouver, BC, Canada

## Abstract

**Background:**

Periodontitis is a chronic inflammatory disease caused by the microbiota of the periodontal pocket. We investigated the association between subgingival bacterial profiles and gene expression patterns in gingival tissues of patients with periodontitis. A total of 120 patients undergoing periodontal surgery contributed with a minimum of two interproximal gingival papillae (range 2-4) from a maxillary posterior region. Prior to tissue harvesting, subgingival plaque samples were collected from the mesial and distal aspects of each tissue sample. Gingival tissue RNA was extracted, reverse-transcribed, labeled, and hybridized with whole-genome microarrays (310 in total). Plaque samples were analyzed using checkerboard DNA-DNA hybridizations with respect to 11 bacterial species. Random effects linear regression models considered bacterial levels as exposure and expression profiles as outcome variables. Gene Ontology analyses summarized the expression patterns into biologically relevant categories.

**Results:**

Wide inter-species variation was noted in the number of differentially expressed gingival tissue genes according to subgingival bacterial levels: Using a Bonferroni correction (p < 9.15 × 10^-7^), 9,392 probe sets were differentially associated with levels of *Tannerella forsythia*, 8,537 with *Porphyromonas gingivalis*, 6,460 with *Aggregatibacter actinomycetemcomitans*, 506 with *Eikenella corrodens *and only 8 with *Actinomyces naeslundii*. Cluster analysis identified commonalities and differences among tissue gene expression patterns differentially regulated according to bacterial levels.

**Conclusion:**

Our findings suggest that the microbial content of the periodontal pocket is a determinant of gene expression in the gingival tissues and provide new insights into the differential ability of periodontal species to elicit a local host response.

## Background

It is well established that the microbiota of the dental plaque are the primary etiologic agents of periodontal disease in humans [1]. The complex consortium of bacteria in the subgingival plaque biofilm [2] is in a state of dynamic equilibrium with the inflammatory response mounted in the adjacent gingival tissues, resulting in interdependent shifts in the composition of both the bacterial community and the ensuing inflammatory infiltrate. Indeed, it is known that microbial profiles of plaques harvested from healthy gingival sulci differ from those stemming from gingivitis or periodontitis lesions [3,4]. Similarly, the cellular and molecular fabric of healthy gingival tissues differs from that of an incipient, early or established periodontitis lesion [5,6].

A relatively new genomic tool that facilitates the study of the biology of cells, tissues or diseases is gene expression profiling, i.e., the systematic cataloging of messenger RNA sequences, and has provided enormous insights in the pathobiology of complex diseases, particularly in cancer research [7,8]. Our group was the first to describe gingival tissue transcriptomes in chronic and aggressive periodontitis [9] and recently provided a comprehensive description of differential gene expression signatures in clinically healthy and diseased gingival units in periodontitis patients [10]. To date, a limited amount of data are available characterizing oral tissue transcriptomes in response to bacterial stimuli. *In vitro *experiments [11-15] demonstrated a degree of specificity in the transcriptional responses of epithelial cells challenged by commensal or pathogenic species and have provided a foundation upon which *in vivo *studies can further contribute [16]. A pilot study including conventionally reared, germ free and SCID mice demonstrated that commensal microbial colonization influences the expression of innate host defense mediators at both the mRNA and the protein level in the periodontal tissues [17]. In a non-oral setting, a number of studies have examined the transcriptional profiles in response to microbial stimuli in intestinal [18-22], gastric [23] and corneal epithelia [24].

In this publication, we expand our earlier work and investigate the association between the subgingival bacterial profile of the periodontal pocket and the whole genome transcriptome of the gingival tissue that is in intimate contact with the microbial biofilm.

## Methods

The study design was approved by the Institutional Review Board of the Columbia University Medical Center.

### Subjects

120 subjects with moderate to severe periodontitis [65 (54.2%) with chronic and 55 with aggressive periodontitis] were recruited among those referred to the Post-doctoral Periodontics Clinic of the Columbia University College of Dental Medicine. Eligible patients were (i) >13 yrs old; (ii) had ≥24 teeth; (iii) had no history of systematic periodontal therapy other than occasional prophylaxis, (iv) had received no systemic antibiotics or anti-inflammatory drugs for ≥6 months, (v) harbored ≥4 teeth with radiographic bone loss, (vi) did not have diabetes or any systemic condition that entails a diagnosis of "Periodontitis as a manifestation of systemic diseases" [25], (vii) were not pregnant, and (ix) were not current users of tobacco products or nicotine replacement medication. Signed informed consent was obtained prior to enrollment.

### Clinical examination

All participants underwent a full-mouth examination of the periodontal tissues at six sites per tooth by a single, calibrated examiner. Variables recorded included presence/absence of visible dental plaque (PL), presence/absence of bleeding on probing (BoP), probing depth (PD), and attachment level (AL). Data were entered chair-side to a computer and stored at a central server.

### Gingival tissue donor areas and tissue sample collection

Subsequently to clinical data entry, a specially developed software identified periodontally 'diseased' and 'healthy' tooth sites based on the clinical data. 'Diseased' sites showed BoP, had interproximal PD ≥4 mm, and concomitant AL ≥3 mm. 'Healthy' sites showed no BoP, had PD ≤4 mm and AL ≤2 mm. Next, the software identified (i) maxillary 'diseased' and 'healthy' interdental papillae, based on the above criteria, and (ii) pairs of diseased interdental papillae with similar clinical presentation (PD and AL within 2 mm of each other). A posterior maxillary sextant encompassing a pair of qualifying 'diseased' interdental papillae was identified.

Periodontal surgery was performed at the identified sextant with no prior supra- or subgingival instrumentation. After local anesthesia, submarginal incisions were performed, mucoperiosteal flaps were reflected, and the portion of each interproximal gingival papilla that adhered to the root surface was carefully dissected. This section comprised the epithelial lining of the interproximal periodontal pockets and the underlying connective tissue. After dissection, the gingival tissue specimens were thoroughly rinsed with sterile normal saline solution and transferred into Eppendorf tubes containing a liquid RNA stabilization reagent (RNA*later*, Ambion, Austin, TX). A minimum of 2 diseased papillae were harvested from each sextant and, whenever available, a healthy tissue specimen was obtained from an adjacent site. After collection of the specimens, pocket elimination/reduction periodontal surgery was completed according to standard procedures. All patients received additional periodontal therapy according to their individual needs.

### RNA extraction, reverse transcription, *in vitro *cRNA synthesis

The tissue specimens were stored in a liquid RNA stabilization reagent (RNA*later*) overnight at 4°C, snap-frozen and stored in liquid nitrogen. All further processing occurred simultaneously for gingival biopsies originating from the same donor. Specimens were homogenized in Trizol (Invitrogen Life Technologies, Carlsbad, CA, USA). After incubation with chloroform and centrifugation at 12,000 g, RNA collected in the upper aqueous phase was precipitated by mixing with 75% isopropyl-alcohol and additional centrifugation and washings. The extracted RNA was purified using a total RNA isolation kit (RNeasy; Qiagen, Valencia, CA, USA), quantified spectrophotometrically, and 7.5 micrograms of total RNA were reverse-transcribed using a one-cycle cDNA synthesis kit (GeneChip Expression 3' amplification one-cycle cDNA synthesis kit; Affymetrix, Santa Clara, CA, USA). Synthesis of biotin-Labeled cRNA was performed using appropriate amplification reagents for in vitro transcription (GeneChip Expression 3'-Amplification Reagents for IVT labeling kit; Affymetrix). The cRNA yield was determined spectrophotometrically at 260 nm. Twenty μg of cRNA were fragmented by incubation in fragmentation buffer at 94°C for 35 min and stored at -80°C until hybridizations.

### Gene Chip hybridizations

Whole genome microarrays (Human Genome U-133 Plus 2.0 arrays; Affymetrix) arrays, comprising 54,675 probe sets to analyze more than 47,000 transcripts including 38,500 well-characterized human genes, were used. Hybridizations, probe array scanning and gene expression analysis were performed at the Gene Chip Core Facility, Columbia University Genome Center. Each sample was hybridized once and each person contributed with 2 to 4 (median 3) tissue samples.

### Harvesting of bacterial plaque

After identification of the interproximal papillae to be harvested and prior to periodontal surgery, subgingival plaque samples were obtained from the mesial and distal aspects of each gingival tissue sample using sterile curettes. After careful removal of supra-gingival plaque, the curette was placed subgingivally until the bottom of the probeable pocket was reached and subgingival plaque was collected by a single scaling stroke. The individual plaque samples were transferred into Eppendorf tubes containing 200 μl of sterile T-E buffer (10 mM Tris HCl, 1.0 mM EDTA, pH 7.6) and were not pooled at any stage of the processing described below.

### Processing of plaque samples

Immediately after transfer to the laboratory the plaque pellet was re-suspended, vigorously vortexed, and 200 μl of a 0.5 M NaOH solution were added. Digoxigenin-labeled, whole genomic probes were prepared by random priming by the use of the High-Prime labeling kit (Roche/Boehringer-Mannheim, Indianapolis, IN, USA) from the following microbial strains: *Aggregatibacter actinomycetemcomitans *(ATCC 43718)*, Porphyromonas gingivalis *(ATCC 33277)*, Tannerella forsythia *(ATCC 43037), *Treponema denticola *(ATCC 35404)*, Prevotella intermedia *(ATCC 25611)*, Fusobacterium nucleatum *(ATCC 10953)*, Parvimonas micra *(ATCC 33270)*, Campylobacter rectus *(ATCC 33238)*, Eikenella corrodens *(ATCC 23834), *Veillonella parvula *(ATCC 10790), and *Actinomyces naeslundii *(ATCC 49340). Further processing was carried out according to the checkerboard DNA-DNA hybridization method [26] as earlier described [27] with the following modifications: The chemiluminescent substrate used for detection was CSPD (Roche/Boehringer-Mannheim). Evaluation of the chemiluminescence signal was performed in a LumiImager F1 Workstation (Roche/Boehringer-Mannheim) by comparing the obtained signals with the ones generated by pooled standard samples containing 10^6 ^or 10^5 ^of each of the species. Standard curves were generated for each species by means of the LumiAnalyst software (Roche/Boehringer-Mannheim), and the obtained chemiluminescent signals were ultimately transformed into bacterial counts and exported into Excel files.

### Statistical Analysis

In all analyses, either R version 2.3.1 (Linux OS) or SAS for PC version 9.1 (SAS Institute, Cary, NC) were used. Gene expression data were normalized and summarized using the log scale robust multi-array analysis (RMA, [28]) with default settings. Laboratory analysis provided a relative quantity of individual bacterial species for each plaque sample by comparison to known standards. Because the distribution of absolute bacterial counts was skewed, values were natural logarithm (ln) transformed, averaged within mouth and standardized by dividing each respective ln(bacterial count) by the population standard deviation for the respective species: one standard deviation on the ln scale (SD_ln_) was treated as equivalent across microbes as previously described [29]. In addition to standardized scores for each individual microbe, we also defined three bacterial groupings ('etiologic burden' (EB), 'putative burden' (PB), and 'health-associated burden (HAB), by summing the standardized values for the various subsets of species as follows [29]: To define EB, we utilized (i) the consensus report of the 1996 World Workshop in Periodontics identifying three bacterial species (*P. gingivalis, T. forsythia *and *A. actinomycetemcomitans*) as causally related to periodontitis [30], and (ii) Socransky's "Red Complex" [31] further identifying *T. denticola *as a species that closely co-varies with *P. gingivalis *and *T. forsythia *in pathological periodontal pockets. The 5 bacterial species deemed putatively associated with periodontal disease (*C. rectus*, *E. corrodens*, *F. nucleatum*, *P. micra *and *P. intermedia*) were grouped as PB [30]. Finally, HAB included two 'health-associated' bacterial species, *A. naeslundii *and *V. parvula *[31].

Differential gene expression was the dependent variable in standard mixed-effects linear regression models which considered patient effects as random with a normal distribution. Standardized bacterial count and gingival tissue status ('healthy' vs. 'diseased') were modeled as fixed effects. Bacterial count was defined as the average value derived from two plaque samples collected from the mesial and distal sites flanking each of harvested papilla, respectively. Gingival tissue status was included in the model to adjust for the confounding effects related to unmeasured characteristics of disease vs. healthy tissue (e.g., tissue properties affecting bacterial colonization or levels of non-investigated bacterial species). To further minimize the potential for confounding, we conducted alternate analyses restricted to diseased tissue and further adjusted for probing depth. Statistical significance for each probe set was determined using both the Bonferroni criterion and q-value [32]. For each probe set, a fold-change was computed by taking the following ratio: raw expression values among gingival tissue samples adjacent to periodontal sites with fifth quintile bacterial colonization levels vs. expression values in samples adjacent to first quintile colonization levels. Therefore, fold-change values represent relative RNA levels in tissues adjacent to 'high' vs. 'low' bacterial colonization sites.

Gene Ontology analysis was performed using ermineJ [33] with the Gene Score Resampling method. P-values generated from the aforementioned mixed-models, were used as input to identify biologically-relevant groups of genes showing differential expression in relation to bacterial colonization. Gene symbols and descriptions were derived from the Gemma System (HG-U133_Plus_2_NoParents.an.zip) and downloaded from http://chibi.ubc.ca/microannots/. Experimental details and results following the MIAME standards [34] are available at the Gene Expression Omnibus (GEO, http://www.ncbi.nlm.nih.gov/geo/) under accession number GSE16134.

### Real-time RT-PCR Confirmations

To independently confirm the expression data generated by the microarray experiments, we performed quantitative real-time RT-PCR analyses for three genes strongly differentially regulated by subgingival bacterial levels (Sperm-associated antigen4 (Spag4), POU class 2 associating factor 1 (POU2AF1), and SLAM family member 7 (SlamF7), while glyceraldehyd-3-phosphatedehydrogenase (GAPDH) was used as a constitutively expressed control gene. In this confirmatory step we used subset of five patients, selected upon the basis of strong differential regulation of the above genes, each contributing with both a 'healthy' and a 'diseased' tissue sample.

In brief, quantitative real-time PCR was performed as described previously [35]. The Taqman Gene Expression Assays Hs00162127_m1, Hs00221793_m1, Hs01573371_m1, and Hs99999905_m1 were used for Spag4, POU2AF1, SlamF7, and glyceraldehyd-3-phosphatedehydrogenase (GAPDH), respectively (Applied Biosystems, Foster City, CA). Three technical replicates per sample and gene were performed. Since in the *Affymetrix *microarray platform, genes are often represented by multiple oligonucleotide probes, we calculated means of the normalized expression data for all probes mapping to the three genes in each gingival tissue specimen. Subsequently, we calculated *Spearman *correlation coefficients for the mean microarray expression values and the Δct values obtained by quantitative RT-PCR.

## Results

The mean age of the enrolled patients was 39.9 years (range 13-76). Sixty one patients (50.8%) were male. Based on self-reported race/ethnicity, 39.2% of the participants were White, 21.7% Black, 27.5% of mixed race, and 73.3% Hispanic.

Among the 310 harvested gingival tissue samples, 69 originated from periodontally healthy sites and 241 from periodontally diseased sites. No healthy tissue samples were available from 51 patients. Probing pocket depth values ranged from 1 to 4 mm in the healthy tissue samples, and between 5 and 11 mm in the diseased tissue samples.

Table 1 describes the subgingival bacterial load in the periodontal pockets adjacent to the 310 harvested gingival tissue samples. As indicated by the > 0 minimum values for all bacteria, all tissue samples were in contact with biofilms that were ubiquitously inhabited by all 11 investigated species. However, the subgingival colonization level by each species varied greatly. Median levels of *P. intermedia, T. forsythia*, and *F. nucleatum *were highest while levels of *A. actinomycetemcomitans, C. rectus *and *V. parvula *were lowest in the pockets adjacent to the obtained gingival tissue specimens.

**Table 1:** Subgingival bacterial load^*a *^in the periodontal pockets adjacent to the harvested gingival tissue samples

Bacterial species	Minimum	**25th pctl**^***b***^	Median	75th pctl	Maximum	AVG	SD
*A. actinomycetemcomitans*	1.4	8.9	18.1	63.6	2306.9	58.5	154.5
*P. gingivalis*	1.1	8.8	112.6	442.0	9740.5	379.3	821.5
*T. forsythia*	3.1	44.4	236.2	758.0	4867.5	543.2	762.9
*T. denticola*	1.6	12.2	70.2	201.3	3318.5	190.8	360.5
*P. intermedia*	4.0	88.2	245.4	531.8	7189.3	470.2	762.7
*F. nucleatum*	14.4	113.1	200.4	348.6	6470.1	270.4	399.5
*P. micra*	4.6	60.7	118.3	211.4	5606.5	189.1	351.9
*C. rectus*	1.1	10.3	28.3	76.3	2457.8	89.1	219.1
*E. corrodens*	1.0	14.3	29.0	71.8	2801.0	74.9	185.6
*V. parvula*	1.5	17.1	35.8	95.2	3004.0	105.1	238.9
*A. naeslundii*	3.8	93.5	179.1	408.3	11353.1	434.4	1003.2

^*a *^Values are bacterial counts × 10 000, obtained through checkerboard DNA-DNA hybridization, and represent the average load of the two pockets adjacent to each tissue sample.

^*b *^Percentile.

Regression models adjusted for clinical status (periodontal health or disease) were used to identify probe sets whose differential expression in the gingival tissues varied according to the subgingival level of each of the 11 investigated species. Using a p-value of < 9.15 × 10^-7 ^(i.e., using a Bonferroni correction for 54,675 comparisons), the number of differentially expressed probe sets in the gingival tissues according to the level of subgingival bacterial colonization was 6,460 for *A. actinomycetemonitans*; 8,537 for *P. gingivalis*; 9,392 for *T. forsythia*; 8,035 for *T. denticola*; 7,764 for *P. intermedia*; 4,073 for *F. nucleatum*; 5,286 for *P. micra*; 9,206 for *C. rectus*; 506 for *E. corrodens*; 3,550 for *V. parvula*; and 8 for *A. naeslundii*. Table 2 presents the top 20 differentially expressed probe sets among tissue samples with highest and lowest levels of colonization (i.e., the upper and the lower quintiles) by *A. actinomycetemcomitans*, *P. gingivalis *and *C. rectus*, respectively, sorted according to decreasing levels of absolute fold change. Additional Files 1, 2, 3, 4, 5, 6, 7, 8, 9, 10, 11 present all the statistically significantly differentially expressed genes for each of the 11 species. Overall, levels of bacteria known to co-vary in the subgingival environment, such as those of the "red complex" [31]) species (*P. gingivalis, T. forsythia*, and *T. denticola*) were found to be associated with similar gene expression signatures in the gingival tissues. Absolute fold changes in gene expression were sizeable among the top 50 probes sets for these three species (range 11.2-5.5 for *P. gingivalis*, 10.4-5.3 for *T. forsythia*, and 8.9-5.0 for *T. denticola*). Corresponding fold changes for the top differentially expressed probe sets ranged between 9.0 and 4.7 for *C. rectus*, 6.9-3.8 for *P. intermedia*, 6.8-4.1 for *P. micra*, 5.8-2.2 for *A. actinomycetemcomitans*, 4.6-2.9 for *V. parvula*, 4.3-2.8 for *F. nucleatum*, 3.2-1.8 for *E. corrodens*, and 2.0-1.5 for *A. naeslundii*. Results for the 'etiologic', 'putative' and 'health-associated' bacterial burdens were consistent with the those for the individual species included in the respective burden scores, and the top 100 probe sets associated with each burden are presented in Additional Files 12, 13, 14.

**Table 2:** Top 20 differentially regulated genes in gingival tissues according to subgingival levels of *A. actinomycetemcomitans, P. gingivalis *and *C. rectus*.

Rank	*A. actinomycetemcomitans*		*P. gingivalis*		*C. rectus*	
	**Gene**^***a***^	**FC**^***b***^	**Gene**	**FC**	**Gene**	**FC**

**1**	hypothetical protein MGC29506	5.76	hypothetical protein MGC29506	11.21	hypothetical protein MGC29506	9.04
**2**	tumor necrosis factor receptor superfamily, member 17	4.23	non-annotated	8.64	non-annotated	7.62
**3**	sperm associated antigen 4	4.01	tumor necrosis factor receptor superfamily, member 17	7.92	tumor necrosis factor receptor superfamily, member 17	6.48
**4**	interferon, alpha-inducible protein 6	3.91	immunoglobulin kappa variable 1-5	7.59	POU domain, class 2, associating factor 1	6.37
**5**	POU domain, class 2, associating factor 1	3.86	non-annotated	7.51	immunoglobulin heavy variable 1-69	6.34
**6**	CD79a molecule, immunoglobulin-associated alpha	3.65	immunoglobulin kappa variable 1-5	7.42	sperm associated antigen 4	6.14
**7**	FK506 binding protein 11, 19 kDa	3.58	immunoglobulin heavy variable 1-69	7.41	KIAA0125	6.10
**8**	hypothetical protein MGC29506	3.56	interferon, alpha-inducible protein 6	7.38	interferon, alpha-inducible protein 6	5.93
**9**	immunoglobulin lambda locus, immunoglobulin lambda constant 1	3.50	POU domain, class 2, associating factor 1	7.18	immunoglobulin kappa constant, immunoglobulin kappa variable 1-5	5.92
**10**	immunoglobulin heavy constant alpha 1	3.47	immunoglobulin kappa variable 1-5	7.16	interferon, alpha-inducible protein 6	5.72
**11**	KIAA0746 protein	3.41	interferon, alpha-inducible protein 6	6.97	immunoglobulin heavy constant alpha 1	5.65
**12**	CD79a molecule, immunoglobulin-associated alpha	3.39	non-annotated	6.96	Fc receptor-like 5	5.60
**13**	family with sequence similarity 46, member C	3.34	immunoglobulin heavy constant alpha 1	6.89	non-annotated	5.55
**14**	non-annotated	3.34	interferon, alpha-inducible protein 6	6.87	interferon, alpha-inducible protein 6	5.53
**15**	interferon, alpha-inducible protein 6	3.26	Fc receptor-like 5	6.85	interferon, alpha-inducible protein 6	5.52
**16**	potassium intermediate/small conductance calcium-activated channel, subfamily N, member 3	3.20	KIAA0125	6.79	immunoglobulin lambda locus, immunoglobulin lambda constant 1 (Mcg marker)	5.49
**17**	immunoglobulin lambda locus, immunoglobulin lambda constant 1 (Mcg marker)	3.16	immunoglobulin kappa variable 1-5	6.70	interferon, alpha-inducible protein 6, immunoglobulin heavy locus (G1m marker)	5.39
**18**	KIAA0746 protein	3.12	immunoglobulin lambda locus	6.67	non-annotated	5.37
**19**	SLAM family member 7	3.11	immunoglobulin lambda locus, immunoglobulin lambda constant 1 (Mcg marker)	6.63	immunoglobulin lambda locus, immunoglobulin lambda constant 1 (Mcg marker)	5.36
**20**	interferon, alpha-inducible protein 6	3.03	sperm associated antigen 4	6.59	immunoglobulin kappa constant, immunoglobulin kappa variable 1-5	5.35

^*a *^Repeated occurrence of the same gene among the top ranked is due to multiple probe sets mapping to the same gene

^*b *^Fold change indicates ratio of expression in gingival tissues in the upper over the lower quintile of colonization by the particular species

Additional regression models utilized data from diseased gingival tissue samples only and included probing pocket depth as an additional continuous covariate. These analyses, despite attenuated p-values and fold changes, confirmed that colonization by specific bacteria remained significantly associated with a differential gene expression in the gingival tissues. For example, in this analysis, among the top 50 differentially expressed probes sets according to colonization levels by *P. gingivalis*, *T. forsythia *or *T. denticola*, the ranges of absolute fold changes were 2.8 - 4.5, 3.3 - 5.5 and 2.5 - 4.3, respectively. All of the top 50 probe sets for each species maintained an FDR<0.05.

Table 3 presents the Spearman correlation coefficients between microarray-generated expression data and Δct values (PCR cycles) of quantitative real-time RT-PCR for three selected genes SPAG4, POU2AF1 and SLAMF7. Since lower Δct values indicate higher levels of expression, the calculated highly negative correlation coefficients between microrray-based expression values and Δct values represent strong and significant positive correlation between data generated by the two platforms.

**Table 3:** Correlation between microarray-based expression data and real time RT-PCR Δct values (PCR cycles) for three genes.

Gene	Spearman correlation coefficient	p-value
Spag4 ^*a*^	-0.95	0.0004
POU2AF1 ^*b*^	-0.94	0.0011
SlamF7 ^*c*^	-0.82	0.0058

^*a *^Sperm-associated antigen 4

^*b *^POU class 2 associating factor 1

^*c *^SLAM family member 7

Gene ontology (GO) analyses identified biological processes that appeared to be differentially regulated in the gingival tissues in relation to subgingival colonization. Additional File 15 provides a complete list of all the statistically significantly regulated GO groups for each of the 11 species. Table 4 exemplifies commonalities and differences in gingival tissue gene expression on the Gene Ontology level with respect to colonization levels by *A. actinomycetemcomitans *and the three "red complex" bacteria. The left column of the Table lists the 20 most strongly differentially regulated GO groups according to levels of *A. actinomycetemcomitans*, while the next three columns indicate the ranking of each particular GO group for *P. gingivalis, T. forsythia *and *T. denticola*, respectively. Although antigen processing and presentation was the highest ranked (i.e., most strongly differentially regulated) GO group for all four species, the second ranked GO group in the *A. actinomycetemcomitans *column (apoptotic mitochondrial changes) was ranked 96^th^, 101^st ^and 96^th^, respectively, for the other three bacteria. Likewise, the fifth ranked group in the *A. actinomycetemcomitans *column (phosphate transport) was ranked 56^th^, 63^rd ^and 71^st^, respectively for the three 3 "red complex" species. Protein-chromophore linkage (ranked 8^th ^for *A. actinomycetemcomitans*) ranked between 147^th ^and 152^nd ^for the other three species. Conversely, second-ranked regulation of cell differentiation for the "red complex" species, ranked 19^th ^for *A. actinomycetemcomitans*.

**Table 4:** Patterns of gene expression in gingival tissues, according to subgingival levels of *A. actinomycetemcomitans, P. gingivalis, T. forsythia *and *T. denticola*.

	*A. actinomycetemcomitans*	*P. gingivalis*	*T. forsythia*	*T. denticola*
**1**	antigen processing and presentation	**1**	**1**	**1**
**2**	apoptotic mitochondrial changes	**96**	**101**	**96**
**3**	antigen processing and presentation of peptide antigen	**3**	**3**	**3**
**4**	antigen processing and presentation of peptide antigen via MHC class I	**4**	**3**	**5**
**5**	phosphate transport	**56**	**63**	**71**
**6**	muscle development	**38**	**39**	**44**
**7**	MAPKKK cascade	**5**	**4**	**7**
**8**	protein-chromophore linkage	**152**	**150**	**147**
**9**	hemopoietic or lymphoid organ development	**9**	**11**	**10**
**10**	hemopoiesis	**11**	**12**	**11**
**11**	immune system development	**8**	**10**	**9**
**12**	protein amino acid N-linked glycosylation	**50**	**81**	**52**
**13**	fatty acid biosynthetic process	**17**	**21**	**8**
**14**	regulation of anatomical structure morphogenesis	**7**	**6**	**7**
**15**	acute inflammatory response	**24**	**18**	**21**
**16**	humoral immune response	**37**	**40**	**35**
**17**	activation of immune response	**62**	**58**	**54**
**18**	regulation of cell adhesion	**51**	**45**	**47**
**19**	regulation of cell differentiation	**2**	**2**	**2**
**20**	hemostasis	**12**	**15**	**14**

The left column lists the top 20 differentially expressed Gene Ontology (GO) groups, according to levels of *A. actinomycetemcomitans *while columns to the right describe the ranking of these particular GO groups for the other three species.

**Figure 1:**
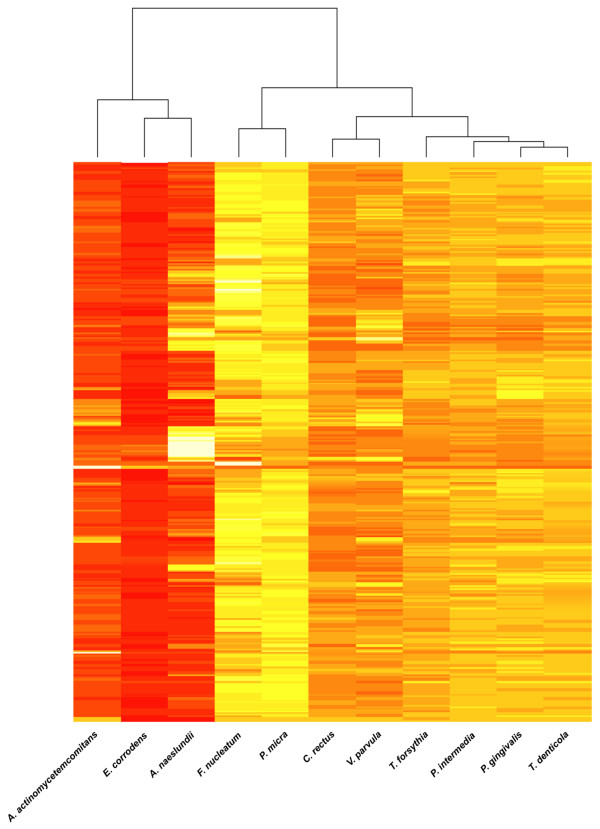
**Cluster analysis of Gene Ontology (GO) groups differentially expressed in gingival tissues according to subgingival colonization by the 11 investigated species**. The clusters identify bacterial species whose subgingival colonization levels are associated with similar patterns of gene expression in the adjacent gingival tissues. The color of the heat map indicates the relative strength of differential regulation of each particular GO group (i.e., each pixel row) among the 11 investigated species, with yellow/white colors indicating strong regulation and red colors weaker regulation.

Figure 1 provides a visual illustration of a cluster analysis that further underscores the level of similarity in gingival tissue gene expression according to colonization by each of the 11 investigated species. The clusters identify bacterial species whose subgingival colonization levels are associated with similar patterns of gene expression in the adjacent gingival tissues. The relative proximity of the investigated species on the x-axis reflects the similarity among the corresponding gingival gene expression signatures. The color of the heat map indicates the relative strength of differential regulation of each particular GO group (i.e., each pixel row) among the 11 species, with yellow/white colors indicating strong regulation and red colors a weaker regulation. Not unexpectedly, "red complex" bacteria clustered closely together, but were interestingly far apart from *A. actinomycetemcomitans*, which showed higher similarity with *E. corrodens *and *A. naeslundii*.

## Discussion

To the best of our knowledge, this is the first study to examine the association between subgingival bacterial colonization patterns and gingival tissue gene expression in human periodontitis. Our data demonstrate that the variable bacterial content of the periodontal pocket correlates with distinct gene expression signatures in the adjacent gingival tissues. Importantly, even though we examined colonization patterns by only a limited number of bacterial species, we found that the variable subgingival bacterial load by several -but clearly not all- species correlated significantly with tissue gene expression. In other words, and to paraphrase both Anton van Leeuwenhoek and George Orwell, our data indicate that all subgingival "animalcules" are not "equal" in this respect.

In a recent publication [10], we presented transcriptomic data from a subset of patients involved in the present report (90 patients and 247 arrays out of the total of 120 patients and 310 arrays included here) and compared gene expression profiles of clinically healthy and diseased gingival tissues in patients with periodontitis. We documented substantial differential gene expression between states of gingival health and disease that was reflected both by genes that were *a priori *anticipated to be variably expressed based on current knowledge (e.g., several inflammatory, immune function- and apoptosis-related genes), but also by genes that are not readily associated with gingival inflammation (e.g., the transcription factor POU2AF1, the sperm associated antigen 4 which appears to be associated with apoptosis (own unpublished data), the cell adhesion-mediating protein desmocollin 1, and the signaling lymphocytic activation molecule family member 7). In the present study, we sought to investigate whether the bacterial content of the periodontal pocket is also a determinant of gene expression in the adjacent gingival tissues in order to enhance our understanding of the host-bacterial interactions that take place in the interface between the plaque biofilm and the periodontal pocket. We realize that the above question can ideally be addressed in a longitudinal prospective rather than a cross-sectional study. Thus, although our analyses considered bacterial colonization as the independent exposure and tissue gene expression as the outcome, it is impossible to rule out reverse causation, i.e., that the qualitative characteristics of the gingival tissue are the determinants of bacterial colonization. However, given that periodontitis is a bacterially-induced infection, the former approach is reasonable in the discussion of the observed correlations between colonization patterns and tissue gene expression signatures. We also want to draw the reader's attention to the fact that, despite our inferences on each particular bacterial species' effect on the gingival tissue transcriptome, we have not studied individual mono-infections. Therefore, any properties ascribed to a particular species with respect to its ability to regulate genes in the gingival tissues cannot be entirely segregated from concomitant synergistic or antagonistic effects of other covarying bacteria among the ones studied or, most importantly, of the several hundreds of cultivable and uncultivable species that are known to colonize the periodontal pocket and were not investigated in this work [36]. Instead, the differential gene expression in the gingival tissues should more appropriately be attributed to the aggregate effect of the mixed microbial burden, and the specific investigated bacteria may simply serve as a surrogate for this mixed microbial burden to which they contribute. It must be further recognized that the gingival tissue transcriptomes are also influenced by a plethora of additional factors beyond those of bacterial origin, including biologically active host-derived molecules and tissue degradation byproducts, that could not be accounted for in our study.

In view of the above, and because the transcriptomic profiles analyzed originate from a mixed cell population comprising gingival epithelial cells, connective tissue fibroblasts and infiltrating cells, our data are not directly comparable with observations from the aforementioned *in vitro *studies of mono-infections of oral epithelial cell lines. Nevertheless, our data corroborate and extent data from these experimental settings. For example, ontology analysis of epithelial cell pathways differentially regulated after infection with *F. nucleatum *[14] identified MAPK signaling and regulation of actin cytoskeleton among the impacted pathways. Likewise, in line with observations by Handfield et al. [11], apoptotic mitochondrial changes, the second highest differentially regulated ontology group according to levels of *A. actinomycetemcomitans *was ranked 96^th ^according to subgingival levels of *P. gingivalis*. Indeed, *A. actinomycetemcomitans *is known to exert strong pro-apoptotic effects on various cell types encountered in inflamed gingival tissues, such as gingival epithelial cells [37] or invading mononuclear cells [38], attributed in part to its potent cytolethal distending toxin [39]. On the other hand, *P. gingivalis *was shown to inhibit apoptosis in primary gingival epithelial cells by ATP scavenging through its ATP-consuming nucleoside diphosphate kinase [40]. In contrast, other *in vitro *studies involving oral epithelial cells (for review see [41]) reported apoptotic cell death induced by *P. gingivalis *at very high (up to 1:50,000) multiplicities of infection [42], which arguably exceeds the *in vivo *burden in the periodontal pocket.

Thus, our data indicate presence of pro-apoptotic alterations in the gingival tissues in *A. actinomycetemcomitans*-associated periodontitis, while the effects of *P. gingivalis *appear to be primarily mediated by other pathways. Interestingly, our data corroborate a recent study that explored the hyper-responsiveness of peripheral blood neutrophils in periodontitis and demonstrated a significantly increased expression of several interferon-stimulated genes [43]. As shown in Table 2, interferon alpha inducible protein-6 was among the top commonly up-regulated genes in gingival tissue lesions according to levels of *A. actinomycetemcomitans*, *P. gingivalis *and *C. rectus*, and tissue-infiltrating neutrophils are a conceivable source for these transcripts.

In general, the magnitude of the differential expression of host tissue genes according to levels of *A. actinomycetemcomitams *(with a total of 68 genes exceeding an absolute fold change of 2 when comparing tissue samples in the upper and lowest quintiles of subgingival colonization; Additional File 1) was more limited than that of bacteria in the 'red complex' (488 genes for *P. gingivalis*, 521 genes for *T. forsythia*, 429 genes for *T. denticola*; Additional Files 2, 3, 4) or *C. rectus *(450 genes; Additional File 8).

The null hypothesis underlying the present study, i.e., that variable subgingival bacterial load by specific bacteria results in no differential gene expression in the adjacent pocket tissues, was rejected by our data. Indeed levels of only 2 of the 11 species investigated appeared to correlate poorly with differential gene expression in the tissues: *A. naeslundii*, whose levels were statistically associated with differential expression of only 8 probe sets out of the approximately 55,000 analyzed, and *E. corrodens *with <1% of the probe sets being differentially regulated between pockets with the highest versus the lowest levels of colonization. In contrast, 15-17% of the examined probes sets were differentially expressed according to subgingival levels of the "red complex" species and *C. rectus*, whose levels were the most strongly correlated with gingival tissue gene expression signatures among all investigated species.

Importantly, the above associations between bacterial colonization and gingival tissue gene expression signatures were confirmed in analyses adjusting for clinical periodontal status, although they were expectedly attenuated. In other words, the difference in the tissue transcriptomes between periodontal pockets with high versus low levels of colonization by the particular species identified as strong regulators of gene expression cannot solely be ascribed to differences in the clinical status of the sampled tissues [10] which is known to correlate well with bacterial colonization patterns [31]. Instead, our analyses based on either statistical adjustment or restriction to 'diseased' tissue samples consistently demonstrate that, even among periodontal pockets with similar clinical characteristics, the subgingival colonization patterns still influence the transcriptome of the adjacent gingival tissues. Assuming a generally positive correlation between gene and protein expression [44,45], this finding is conceptually important as it suggests that the 'phenotype' of the periodontal pocket, and by extension its potential to experience additional periodontal tissue breakdown and/or an unfavorable or favorable treatment response, is also dependent on its bacterial content, and not merely on the traditional clinical parameters (non-specific plaque accumulation, bleeding on probing, probing depth and attachment level). It also provides biology-founded ammunition in favor of the controversial argument that microbial diagnostics have a place in the decision-making and therapeutic management of patients with periodontitis [46].

Finally, we emphasize that the subject sample involved in the present study included both chronic and aggressive periodontitis patients and subjectsbelonging to various race/ethnicity groups. It is conceivable that the typeof disease and race/ethnicity-related charactersitics may be additional determinants of the gingival tissue transcriptome and/or may act asmodifiers of the association between bacterial colonization patterns andtissue gene expression. We intend to explore these possibilities insubsequent reports.

## Conclusion

Using data from 120 patients, 310 gingival tissue samples and the adjacent 616 subgingival plaque samples, we demonstrate a strong correlation between the bacterial content of the periodontal pocket and the gene expression profile of the corresponding gingival tissue. The findings indicate that the subgingival bacterial load by several - but clearly not all - investigated periodontal species may determine gene expression in the adjacent gingival tissues. These cross-sectional observations may serve as a basis for future longitudinal prospective studies of the microbial etiology of periodontal diseases.

## Authors' contributions

PNP conceived of the study, is the Principal Investigator of the grant that provided the funding, and authored the manuscript; JHB and DLW recruited and treated the patients, and harvested the microbial and gingival tissue samples; MK carried out the laboratory work for the gene expression assessments and RC for the microbiological assessments; RD carried out the gene expression analysis and assisted in the authorship of the manuscript; MH and PP assisted in the data analysis and the authorship of the manuscript. All authors read and approved the finalized text.

## Supplementary Material

Additional file 1 **Table S1**. Statistically significantly differentially expressed probe sets in the gingival tissues according to levels of *A. actinomycetemcomitans *in the adjacent pockets. Click here for file

Additional file 2 **Table S2**. Statistically significantly differentially expressed probe sets in the gingival tissues according to levels of *P. gingivalis *in the adjacent pockets. Click here for file

Additional file 3 **Table S3**. Statistically significantly differentially expressed probe sets in the gingival tissues according to levels of *T. forsythia *in the adjacent pockets. Click here for file

Additional file 4 **Table S4**. Statistically significantly differentially expressed probe sets in the gingival tissues according to levels of *T. denticola *in the adjacent pockets. Click here for file

Additional file 5 **Table S5**. Statistically significantly differentially expressed probe sets in the gingival tissues according to levels of *P. intermedia *in the adjacent pockets. Click here for file

Additional file 6 **Table S6**. Statistically significantly differentially expressed probe sets in the gingival tissues according to levels of *F. nucelatum *in the adjacent pockets. Click here for file

Additional file 7 **Table S7**. Statistically significantly differentially expressed probe sets in the gingival tissues according to levels of *P. micra *in the adjacent pockets. Click here for file

Additional file 8 **Table S8**. Statistically significantly differentially expressed probe sets in the gingival tissues according to levels of *C. rectus *in the adjacent pockets. Click here for file

Additional file 9 **Table S9**. Statistically significantly differentially expressed probe sets in the gingival tissues according to levels of *E. corrodens *in the adjacent pockets. Click here for file

Additional file 10 **Table S10**. Statistically significantly differentially expressed probe sets in the gingival tissues according to levels of *V. parvula *in the adjacent pockets. Click here for file

Additional file 11 **Table S11**. Statistically significantly differentially expressed probe sets in the gingival tissues according to levels of *A. naeslundii *in the adjacent pockets. Click here for file

Additional file 12 **Table S12**. A list of the top 100 differentially expressed probe sets in the gingival tissues according to levels of 'Etiologic burden' in the adjacent pockets. Click here for file

Additional file 13 **Table S13**. A list of the top 100 differentially expressed probe sets in the gingival tissues according to levels of 'Putative burden' in the adjacent pockets. Click here for file

Additional file 14 **Table S14**. A list of the top 100 differentially expressed probesets in the gingival tissues according to levels of 'Health-associated burden' in the adjacent pockets. Click here for file

Additional file 15 **Table S15**. List of all statistically significantly regulated GO groups in the gingival tissues according to levels of each of the 11 investigated species in the adjacent pockets. Click here for file
